# Anti-idiotypic Antibodies against BP-IgG Prevent Type XVII Collagen Depletion

**DOI:** 10.3389/fimmu.2017.01669

**Published:** 2017-11-27

**Authors:** Mayumi Kamaguchi, Hiroaki Iwata, Yuiko Mori, Ellen Toyonaga, Hideyuki Ujiie, Yoshimasa Kitagawa, Hiroshi Shimizu

**Affiliations:** ^1^Department of Dermatology, Hokkaido University Graduate School of Medicine, Sapporo, Japan; ^2^Department of Oral Diagnosis and Medicine, Graduate School of Dental Medicine, Hokkaido University, Sapporo, Japan

**Keywords:** bullous pemphigoid, type XVII collagen, intravenous immunoglobulin, idiotypic antibody, depletion, autoantibody

## Abstract

Bullous pemphigoid (BP) mainly targets type XVII collagen (COL17). Intravenous immunoglobulin (IVIg) is used to treat numerous autoimmune diseases, including BP. The major mechanism of action for IVIG is thought to be its immunomodulatory effect. However, little is known about the precise mechanisms of IVIg in BP. We investigate the cellular effects of IVIg, toward treatments for BP. Keratinocytes were treated with IgG from BP patients (BP-IgG) and with IVIg, and then the COL17 expression was detected by Western blotting. Cell adhesion and *ex vivo* dermal–epidermal separation were also investigated for the condition with BP-IgG and IVIg. BP-IgG targeting the non-collagenous 16A domain induces the depletion of COL17 in cultured keratinocytes (DJM-1 cells). The COL17 levels in DJM-1 cells were decreased by 50% after 4 h of BP-IgG stimulation as determined by Western blotting. By contrast, BP-IgG with IVIg was found to result in 70–90% increases in COL17 and to restore adhesion to the plate. Interestingly, IVIg significantly inhibited the binding of BP-IgG to the COL17-enzyme-linked immunosorbent assay plate, and this was due to anti-idiotypic antibodies against BP-IgG. When anti-idiotypic antibodies against BP-IgG in 0.02% of IVIg were depleted from IVIg, those antibodies did not exhibit inhibitory effects on COL17 depletion. When cryosections of human skin were incubated with BP-IgG in the presence of leukocytes, dermal–epidermal separation was observed. BP-IgG treatment with IVIg or anti-idiotypic antibodies did not induce such separation. These findings strongly suggest the presence of anti-idiotypic antibodies against anti-COL17 IgG in IVIg. This mechanism of IVIg could be a target for therapies against BP.

## Introduction

The first uses of intravenous immunoglobulin (IVIg) were in immunodeficient individuals and individuals with severe infections. IVIg is currently used to treat numerous autoimmune diseases, including rheumatoid arthritis, systemic lupus erythematosus, and autoimmune blistering diseases (AIBDs) (1). Controlled studies of IVIg as a treatment for pemphigus and pemphigoid patients found IVIg to be a safe, effective treatment (2, 3). Furthermore, several case reports have described the use of IVIg to treat AIBDs such as pemphigoid and epidermolysis bullosa acquisita (EBA) (4–6). Although various modes of action for IVIg have been proposed in AIBDs, the mechanisms behind its effect are still not fully understood (7, 8). The major mechanism of action for IVIg in AIBDs is thought to be its immunomodulatory effect (1, 9–11). In addition, anti-idiotypic antibodies against pathogenic antibodies have been reported in autoimmune disorders (1, 12). Although there are many autoimmune disorders, anti-idiotypic antibodies against autoantibodies have been proved in only several autoimmune disorders (13–18).

Bullous pemphigoid (BP) is the most common AIBD (19). Two autoantigens, type XVII collagen (COL17, also called BP180) and BP230, which form a hemidesmosome, are targeted in BP, and COL17 is particularly relevant to the pathogenesis (19). Antibody-induced tissue damage is a major pathology in autoantibody-mediated autoimmune diseases (20). The activation of complements and/or inflammatory cells, including neutrophils and eosinophils, is crucial to the development of clinical phenotypes in animal models (21–23). In addition, molecular or cellular mechanisms have been proposed. IgG from BP patients (BP-IgG) targeting the non-collagenous 16A (NC16A) domain of COL17 induces the depletion of COL17 in cultured keratinocytes (24). It is thought that the shortage of COL17 causes an insufficiency of hemidesmosomes during remodeling that eventually results in weak cell adhesion to the basement membrane (25).

Regarding treatments for BP patients, it has been reported recently that IVIg provides therapeutic benefits to BP patients (3). A randomized, double-blind, placebo-controlled clinical study concluded that IVIg has therapeutic benefits for patients with BP who are resistant to systemic steroid therapy. The inhibition of autoantibody production and inflammatory cascades are major strategies in BP treatment. Prednisolone is thought to have dual effects and is commonly used. In most BP treatments, the targets are immune cells, including neutrophils and antigen-specific B cells and/or T cells. However, little is known about the effects of IVIg in keratinocytes expressing the autoantigens. This study focused on the cellular effects of IVIg, for the treatment of BP.

## Materials and Methods

### BP Patients and Total IgG Purification

The BP patients fulfilled both inclusion criteria: (i) clinical blistering or erosions on the skin and (ii) circulating autoantibodies against COL17 as detected by BP180-NC16A enzyme-linked immunosorbent assay (ELISA)/CLEIA (MBL, Nagoya, Japan). BP-IgG was purified from plasma obtained by apheresis in a severe BP patient. Total IgG was purified using a protein G affinity column according to the manufacturer’s instructions (GE Healthcare, Amersham, UK). In accordance with the Hokkaido University Hospital bylaws and standard operating procedures approved by the Hokkaido University Hospital Review Board, we obtained patient consent for experimental procedures to be performed at Hokkaido University Hospital. A full review and approval by an ethics committee were not required, according to local guidelines. The studies were conducted in accordance with the Helsinki guidelines.

### Anti-COL17 NC16A IgG Purification

Anti-COL17 NC16A IgG was purified from total IgG using a protein G affinity column by means of the HiTrap HNS-activated HP column according to the manufacturer’s instructions (GE Healthcare). Briefly, GST fusion COL17 NC16A was produced as previously described (26). The recombinant protein was coupled with the HiTrap NHS-activated HP column. The titer of anti-COL17-specific IgG was measured by indirect immunofluorescent staining. Indirect immunofluorescent staining using anti-COL17-specific IgG (concentration 0.1 mg/ml) demonstrated titers greater than 1:32,000.

### Treatment Agents

Two different IVIgs (Nihon Pharmaceutical Co., Ltd., Tokyo, Japan, and Baxter International Inc., Deerfield, IL, USA; diluted with PBS) were purchased and used for this study. The concentrations of each agent for the treatment are given in Section “Results” and the figure legends.

### Depletion of Anti-idiotypic Antibodies

To deplete anti-idiotypic antibodies against anti-COL17 NC16A IgG, anti-COL17 NC16A IgG was coupled to a HiTrap NHS-activated HP column according to the manufacturer’s instructions (GE Healthcare). IVIg was passed through the column to remove anti-idiotypic antibodies, and then flow-through fractions (the “IVIg-depleted” sample) and the elution fraction (the “idiotype” sample) were used for the depletion assay. To evaluate the depletion efficacy, 96-well microtiter plates (Maxisorp; Nunc, Roskilde, Denmark) were coated with purified anti-COL17 NC16A IgG (500 ng/well), normal human IgG and PBS. Nonspecific binding was reduced by blocking with protein-free blocking buffer (Thermo Fisher Scientific, Rockford, IL, USA) at room temperature (RT) for 1 h. Plates were subsequently incubated with biotin-conjugated IVIg (1 mg/ml). IVIg was conjugated with biotin using a biotin labeling kit according to the manufacturer’s instructions (Dojindo, Kumamoto, Japan). Finally, plates were incubated with HRP-conjugated streptavidin (Thermo Fisher Scientific) for IgG subclasses at RT for 0.5 h.

### Cell Culture

DJM-1 cells isolated from human skin squamous cell carcinoma (27) were cultured in DMEM. To investigate the depletion of COL17, cells were cultured to approximately 40% confluence (24). DJM-1 cells were pretreated with agents for 1 h, and then BP-IgG (concentration 1 mg/ml) was added to the culture media for 4-h incubation. In some experiments, DJM-1 cells were treated with BP-IgG for 4 h followed by IVIg for 1 h.

### Western Blotting

For Western blot analysis of whole-cell lysates, cells were lysed in RIPA buffer (Thermo Fisher Scientific) containing a protease inhibitor cocktail (Sigma Aldrich), and the lysates were centrifuged. Each fraction was subjected to SDS–PAGE in 6% polyacrylamide gel. The gels were transferred onto nitrocellulose membranes. Blotting was performed using rabbit anti-COL17 (1:2,000 dilution) (26), rabbit anti-β-tubulin (Abcam, Tokyo, Japan, 1:20,000 dilution), and anti-integrin α6 (Santa Cruz, Dallas, TX, USA, 1:500 dilution) as the primary antibodies, followed by incubation with HRP-conjugated goat anti-rabbit or anti-mouse IgG (Life Technologies, 1:5,000 dilution). Signals were visualized with Clarity Western ECL Substrate (Bio-Rad Laboratories, Hercules, CA, USA).

### Enzyme-Linked Immunosorbent Assay

BP180-NC16A ELISA was performed according to the manufacturer’s instructions with minor modifications (MBL). Briefly, the ELISA plate was incubated with 100 µl of IVIg (5 mg/ml), bovine serum albumin (BSA, 5 mg/ml), or PBS. Subsequently, the plate was incubated with 1 mg/ml BP-IgG with/without PBS washing. The ELISA index value was calculated according to the manufacturer’s instructions. ELISA using sera from BP patients and healthy volunteers was performed without washing the plate after IVIg incubation.

### Cell Adhesion Test

BP-IgG stimulation leads to reductions in COL17 amount and in cell adhesion to the culture plate (24). Cells were placed on a vortex mixer for 20 min after BP-IgG stimulation with/without IVIg pretreatment. The adhesion of DJM-1 cells to the bottom of the culture plate was assayed by determining the number of adherent cells after vibration. After PBS washing, cells that remained on the bottom of the culture plate were treated with 0.25% trypsin for 5 min at 37°C. The released cells were counted using a blood cell counter under a microscope.

### *Ex Vivo* Dermal–Epidermal Separation Assay (Cryosection Assay)

*Ex vivo* autoantibody-induced, neutrophil-dependent dermal–epidermal separation was performed as described (28, 29). Briefly, 5-µm cryosections from normal human skin were incubated with BP-IgG (2 mg/ml) in the presence or absence of 5 mg/ml IVIg at 37°C for 1 h. After washing with PBS, the slides were covered with a second slide that was taped at each end to form a chamber. Subsequently, 10^7^ cells/ml of freshly isolated normal human leukocyte suspension was injected into the chamber and incubated at 37°C for 5 h. Sections were subsequently stained with H&E. We tested the experiments using two different blood donors.

### Statistical Analyses

Statistical calculations were performed using SigmaPlot (Version 12.0, Systat Software, Chicago, IL, USA). To compare the parameters in the COL17-depletion assay and COL17-ELISA, one-way ANOVA test was used. A comparison of the COL17-ELISA using patient sera with/without IVIg was performed using the Wilcoxon signed-rank test. A *p*-value of <0.05 was considered statistically significant. The graphs present the median ± SD.

## Results

### IVIg Prevents COL17 Depletion of Keratinocytes Induced by BP-IgG

**Figure 1:**
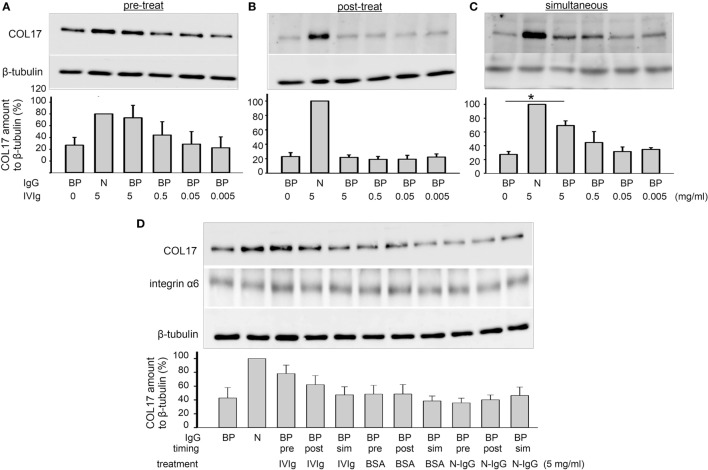
Intravenous immunoglobulin (IVIg) prevents BP-IgG-induced type XVII collagen (COL17) depletion. DJM-1 cells (40% confluency) were treated with 1.0 mg/ml BP-IgG. Western blotting using total cell lysate was performed for anti-COL17. **(A)** DJM-1 cells were treated with IVIg (5–0.5–0.05–0.005 mg/ml) for 1 h followed by BP-IgG for 4 h (pretreatment). **(B)** DJM-1 cells were treated with BP-IgG for 4 h followed by IVIg for 1 h (posttreatment). **(C)** BP-IgG and IVIg were incubated in advance, and then added to the culture medium together (simultaneous). **(D)** DJM-1 cells were treated with appropriate controls. The amount of integrin α6, which is a transmembrane protein and a hemidesmosome component, was not changed. The representative Western blot is presented. Each experiment was performed at least three times, with *p* < 0.05. BP, BP-IgG; N, normal human IgG.

When DJM-1 cells are treated with BP-IgG, the amount of COL17 is decreased as determined by Western blotting (COL17-depletion assay) (24). In this study, we examined the effects of IVIg by means of a COL17-depletion assay. For a COL17-depletion assay, 40% confluent cells were incubated with BP-IgG (concentration: 1 mg/ml) for 4 h. The amount of COL17 relative to β-tubulin was determined by Western blotting. DJM-1 cells were treated with BP-IgG before (the pretreatment sample) or after (the posttreatment sample) the addition of serially diluted IVIg. BP-IgG without IVIg induced an approximately 50–60% reduction of COL17 in DJM-1 cells. By contrast, BP-IgG pretreated with 5 mg/ml IVIg restored the amount of COL17 by 70–90% (*p* < 0.05, Figure 1A). IVIg posttreatment after 4 h of BP-IgG stimulation did not influence COL17 depletion (Figure 1B). Given that IVIg has direct effects on keratinocytes, such as on caspase expression (30), we incubated BP-IgG with IVIg in advance and then added them to the culture medium together (simultaneous sample). The COL17 depletion was restored similar to that in Figure 1A (Figure 1C). The amount of integrin α6, which is a transmembrane protein that is a hemidesmosomal component, was unchanged (Figure 1D). The same concentration (5 mg/ml) of BSA and normal human IgG did not influence the amount of COL17 under any conditions (Figure 1D).

The highest concentration of IVIg (5 mg/ml) was designed based on the estimated serum concentration with standard daily IVIg treatment (0.4 g/kg/day) (3).

### IVIg Restores the Loss of Cell Adhesion That Is Induced by BP-IgG

**Figure 2:**
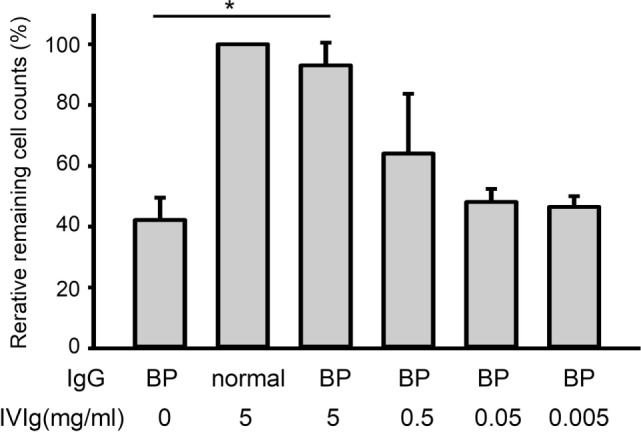
Intravenous immunoglobulin (IVIg) restores the loss of cell adhesion induced by BP-IgG. DJM-1 cells were treated with 1.0 mg/ml BP-IgG. Culture plates were placed on a vortex for 20 min. After PBS washing, cells that remained on the bottom of the culture plate were counted using a blood cell counter under a microscope. The experiment was performed three times, with *p* < 0.05.

Furthermore, cell adhesion was found to be reduced by approximately 60% under BP-IgG stimulation (Figure 2). Pretreatment with IVIg restored cell adhesion. Cells remaining in the culture plate were significantly increased with 5 mg/ml IVIg pretreatment (*p* < 0.05, Figure 2). Pretreatment with 5 mg/ml IVIg restored adhesion by 90% compared with normal IgG stimulation.

### IVIg Prevents BP-IgG Binding to COL17

**Figure 3:**
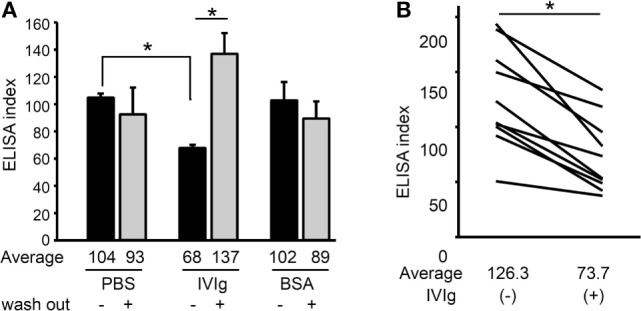
Intravenous immunoglobulin (IVIg) prevents BP-IgG from binding to type XVII collagen (COL17). **(A)** BP-IgG titers were measured using a COL17-non-collagenous 16A enzyme-linked immunosorbent assay (ELISA) in the presence of IVIg and bovine serum albumin (BSA). The ELISA plates were washed with PBS after IVIg pretreatment to remove IVIg. Each experiment was performed four times. **(B)** The ELISA indexes of 10 BP patients’ sera were measured with/without IVIg. Each experiment was performed three times, with *p* < 0.05.

We subsequently investigated the mechanism of action of IVIg in this COL17-depletion assay. The titer of BP-IgG used for the COL17-depletion assay was measured with IVIg (5 mg/ml), BSA (5 mg/ml) or PBS by ELISA. ELISA plates were preincubated with either IVIg or BSA, and BP-IgG was subsequently added. To rule out the possibility of IVIg masking the antigen, the ELISA plate was washed with PBS after IVIg pretreatment, and then BP-IgG was added. Interestingly, 5 mg/ml IVIg (without washing) significantly inhibited the binding of BP-IgG to the ELISA plate (*p* < 0.05, Figure 3A). IVIg pretreatment (without washing) reduced the average titer by 30% compared with PBS pretreatment (Figure 3A, black bar). This inhibition was not observed using IVIg (with washing) or the same concentration of BSA. These results suggest that IVIg impairs BP-IgG’s ability to bind to COL17, in which IVIg may function as an anti-idiotypic antibody. To confirm our results, samples from 10 BP patients were evaluated in the same manner. The serum titers for all 10 patients were found to be reduced by approximately 40% in the presence of IVIg (without washing) (*p* < 0.05, Figure 3B).

### IVIg Contains Anti-idiotypic Antibodies against Anti-COL17 IgG

**Figure 4:**
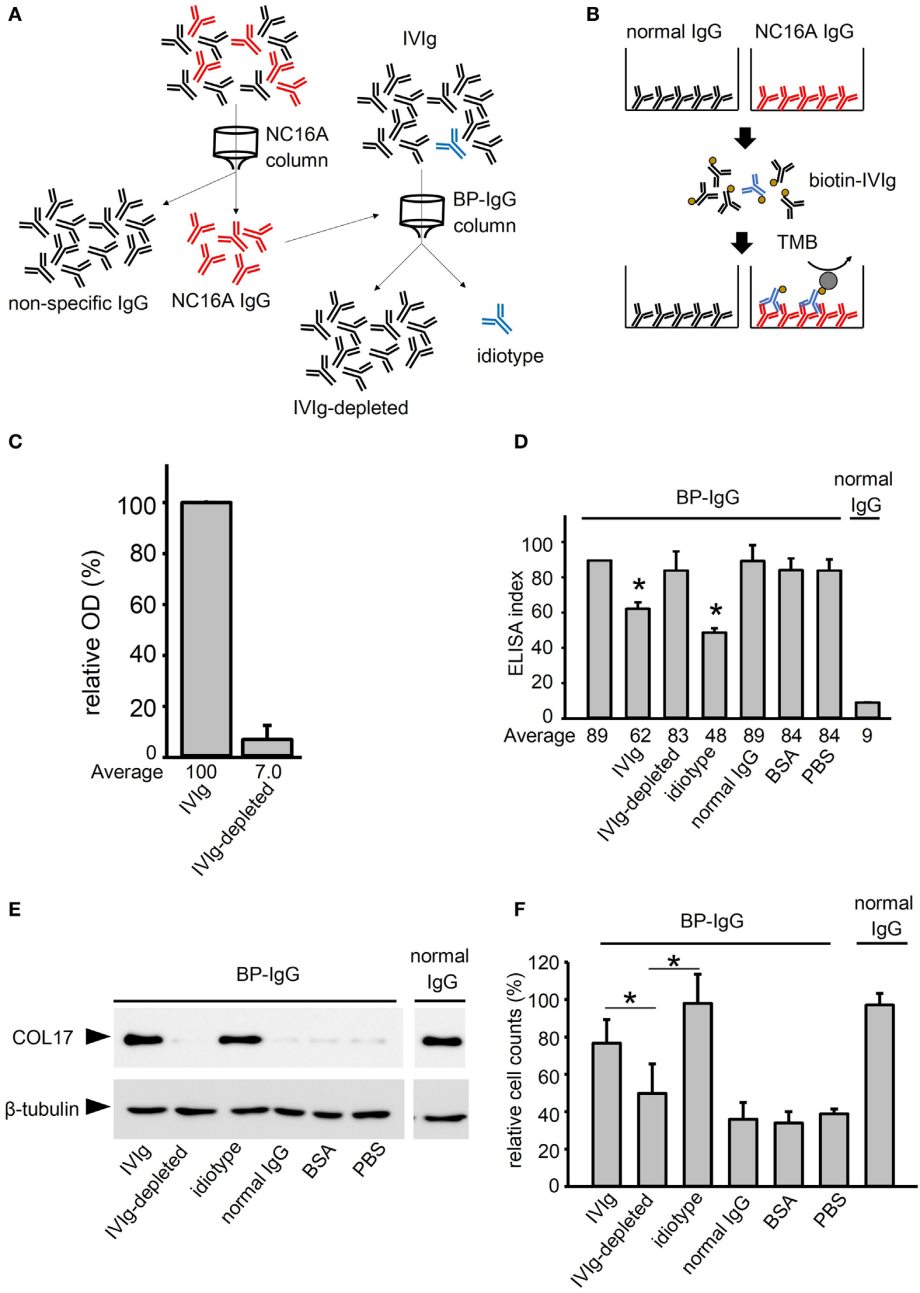
Intravenous immunoglobulin (IVIg) contains anti-idiotypic antibodies against anti-type XVII collagen (COL17) IgG. **(A)** To deplete idiotypic antibodies, anti-COL17 non-collagenous 16A (NC16A) IgG was purified. Next, anti-COL17 NC16A IgG was coupled to a column. IVIg was passed through the column, and then the flow-through fraction (IVIg depleted) and the elution fraction (idiotype) were corrected. **(B)** To evaluate the depletion efficacy, 96-well microtiter plates were coated with anti-COL17 NC16A IgG and normal human IgG (500 ng/well). The plates were incubated with biotin-conjugated IVIg (1 mg/ml; idiotype sample: 0.1 mg/ml). Finally, the plates were incubated with HRP-conjugated streptavidin. The depletion efficacy was calculated as follows: $\frac{\left(\text{IVIg-depleted OD to anti-COL17 NC16A IgG}\right)–\left(\text{IVIg OD to normal IgG}\right)}{\left(\text{IVIg OD to anti-COL17 NC16A}\right)–\left(\text{IVIg OD to normal IgG}\right)}\times 100$. **(C)** To calculate the depletion efficacy, the relative OD score of biotin-conjugated IVIg (5 mg/ml) against anti-COL17 NC16A IgG was determined. Using IVIg samples, the following were performed: **(D)** COL17 NC16A ELISA, **(E)** COL17-depletion assay, and **(F)** cell adhesion test. Bovine serum albumin (BSA) and normal human IgG at the same concentrations were used as controls. Data are based on duplicate samples, and each experiment was performed three times, with *p* < 0.05.

Next, to confirm that anti-idiotypic antibodies were actually present in the IVIg, we performed experiments to identify them directly. Anti-idiotypic antibodies in the IVIg were depleted by anti-COL17 NC16A IgG (IVIg, IVIg depleted, and idiotype fractions, Figure 4A). IVIg contained approximately 0.02% of anti-idiotypic antibodies against COL17 IgG (Figure 4B). The depletion efficiency was more than 90% (Figure 4C). The titers of BP-IgG were measured in the presence of IVIg (IVIg 5 mg/ml and idiotype 2.6 µg/ml) and normal IgG (5 mg/ml). The titers were found to be decreased in the setting of BP-IgG with IVIg and idiotype, but not BP-IgG with IVIg depleted (Figure 4D). Next, a COL17-depletion assay was performed using IVIg (5 mg/ml) and anti-idiotypic antibodies (2.6 µg/ml). IVIg depleted did not show blocking effects on COL17-depletion (Figure 4E). Furthermore, adhesion was also tested using IVIg and anti-idiotypic antibodies. IVIg depleted did not restore the adhesive strength compared with IVIg and idiotype (Figure 4F).

### IVIg Mitigates BP-IgG-Induced Dermal–Epidermal Separation on Cryosections

**Figure 5:**
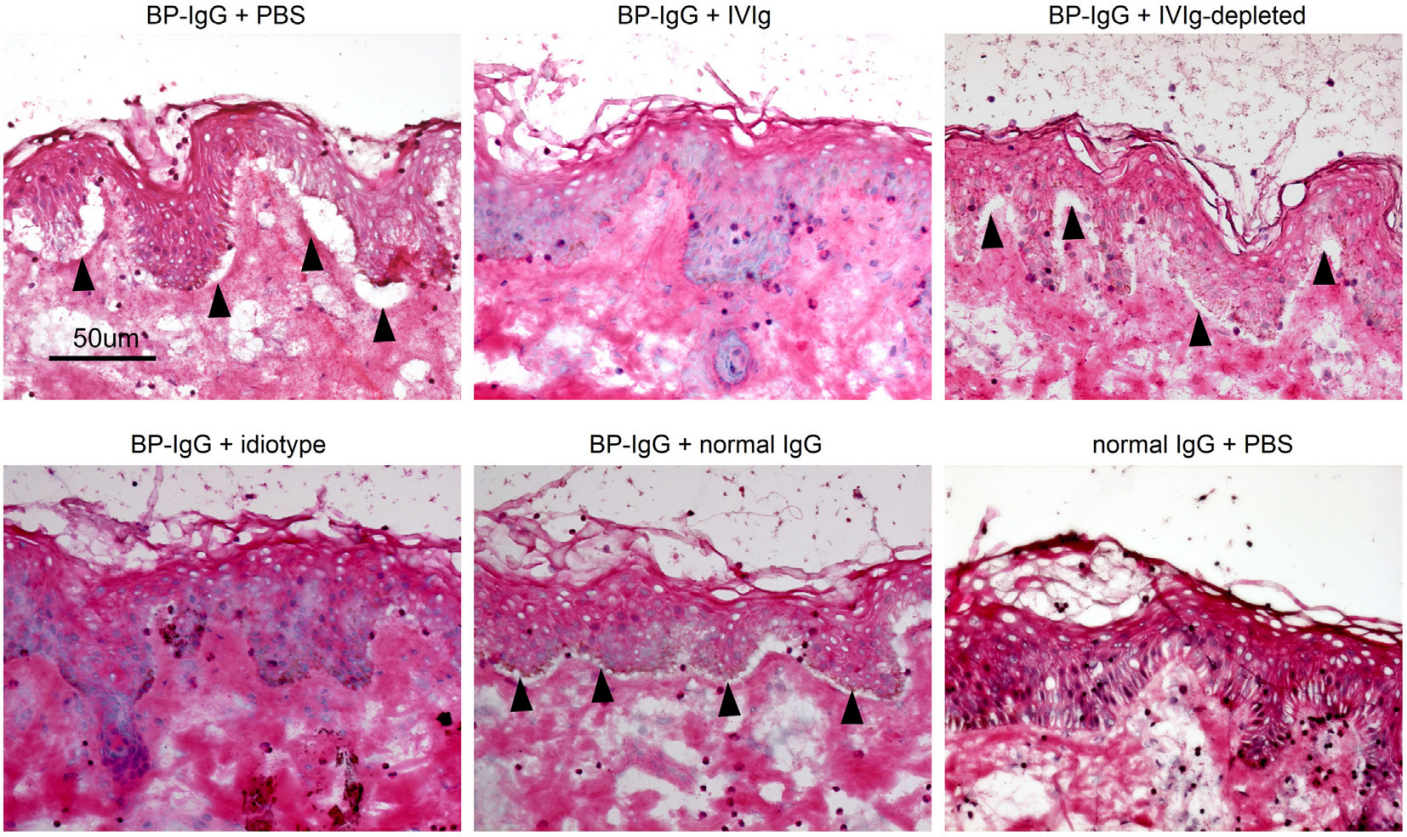
Intravenous immunoglobulin (IVIg) prevents dermal–epidermal separation *ex vivo*. Cryosections of human skin were incubated with BP-IgG in the presence of IVIg (5 mg/ml, idiotype 2.6 µg/ml) and normal human IgG (5 mg/ml) for 1 h. After washing with PBS, sections were incubated with freshly isolated human leukocytes (1 × 10^7^ cells/ml) for 5 h. Arrows indicate dermal–epidermal separation. Each experiment was performed three times. Representative results for each setting are presented.

According to our results, the therapeutic effects of IVIg potentially involve anti-idiotypic antibodies. We next examined the blocking effects of IVIg using an *ex vivo* assay, demonstrating the dermal–epidermal separation induced by BP-IgG in the presence of leukocytes (28, 29). In case of BP-IgG with PBS, dermal–epidermal separation was observed 5 h after incubation with normal human leukocytes (Figure 5, upper left). Next, we added IVIg, BSA and normal human IgG and incubated them with BP-IgG on the cryosections for 1 h, followed by incubation with normal human leukocytes for 5 h. IVIg depleted (5 mg/ml, upper right) and normal human IgG (5 mg/ml, lower middle) did not block the dermal–epidermal separations. By contrast, IVIg (5 mg/ml, upper middle) and the idiotype (2.6 µg/ml, lower left) protected the dermal–epidermal separation.

### The Results Were Reproduced Using a Different Company’s Product

**Figure 6:**
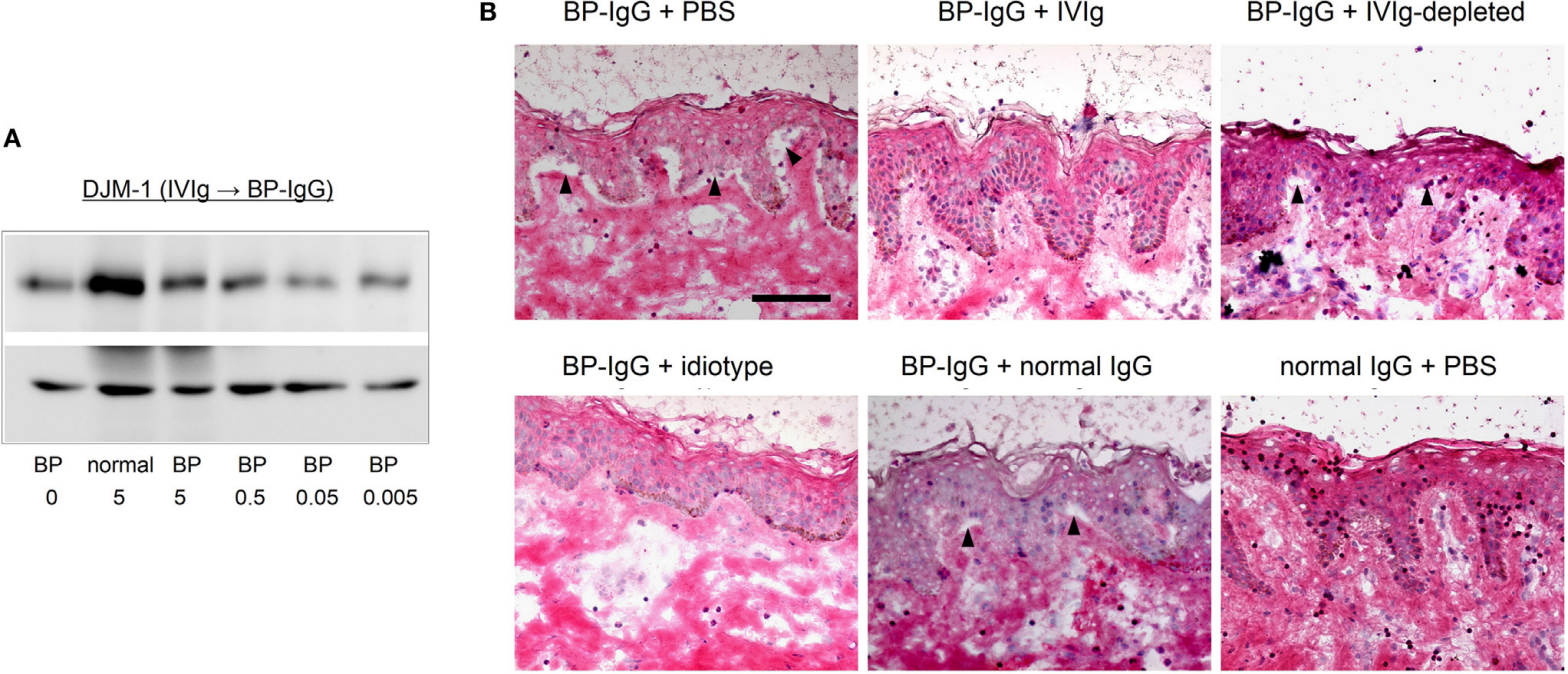
A different company’s intravenous immunoglobulin (IVIg) reproduces the results. Using IVIg from a different company, the type XVII collagen (COL17)-depletion assay and *ex vivo* assay were performed. **(A)** COL17-depletion assay using IVIg from a different company (pretreatment). **(B)** *Ex vivo* assay using IVIg from a different company.

There is great concern about differences arising from differences in IVIg lots or in companies. Therefore, we tested an IVIg from the different company. An IVIg product from this different company was found to prevent the COL17 depletion of keratinocytes induced by BP-IgG (Figure 6A). Furthermore, we found the same blocking effects of IVIg using an *ex vivo* assay (Figure 6B).

## Discussion

Intravenous immunoglobulin therapy is currently applied in autoimmune diseases, and various mechanisms of action for IVIg are involved. Previous clinical studies concluded that IVIg therapy for AIBD is a safe, effective strategy (2, 3, 31–34). The modes of action of IVIg are divided into two major mechanisms (1). One mechanism involves the F(ab)2 fragment, which is responsible for antigen recognition. The other mechanism involves the Fc fragment, which contributes to effector cell activation. Anti-idiotypic antibodies work *via* the F(ab)2 fragment, which neutralize the pathogenic antibodies in several autoimmune disorders (1, 12). However, until now, there have been no reports on anti-idiotypic antibodies in BP. According to our COL17-depletion results, we expected IVIg to contain anti-idiotypic antibodies against BP-IgG and to prevent BP-IgG binding to autoantigen COL17. We clearly demonstrated the presence of very small amounts of anti-idiotypic antibodies against anti-COL17 IgG (0.02%) in IVIg. Even very small amounts of anti-idiotypic antibodies can prevent COL17 depletion. In addition, after the depletion of anti-idiotypic antibodies from IVIg, the ability of IVIg to block COL17 depletion was not observed. IVIg is purified from serum pooled from healthy volunteers; therefore, the IVIg used in this study may have contained anti-idiotypic antibodies to BP-IgG by chance. However, we ruled this out by reconfirming similar results using IVIg from a different company.

Although there are several lines of clinical evidence, the precise mechanisms of IVIg have yet to be fully elucidated in AIBD. Li et al. reported that IVIg therapy inhibited an experimental model of AIBD by accelerating the degradation of pathogenic IgG (9). This inhibitory effect of IVIg in experimental BP was completely dependent on the neonatal Fc receptor (FcRn) *via* the Fc fragment. FcRn is associated with the half-life of IgG, and IgG recycled *via* FcRn increases that half-life (1). In an experimental model of EBA, IVIg exhibited therapeutic effects similar to those seen in systemic steroid therapy (10). In this EBA model, the disease was associated with neutrophil activation *via* the Fc gamma receptor (FcgR) IV (35). Interestingly, IVIg treatment was found to reduce circulating autoantibodies and to modulate FcgRIV expression on neutrophils (10). FcgR modulation on neutrophils is also mediated by the Fc fragment of IVIg (1).

Systemic prednisolone is the most common and the most recommended therapy for BP (19, 36, 37). Long-term prednisolone administration is associated with several risks, such as diabetes mellitus, infections, and osteoporosis. By contrast, IVIg is a safe, useful treatment for severe or high-risk cases, e.g., immunocompromised individuals or those with chronic viral infections, because it is less immunosuppressive. Although anti-idiotypic antibodies have been reported in several autoimmune disorders, a disease-specific therapy using anti-idiotypic antibodies has not been established. One major difficulty is that patients have polyclonal autoantibodies. Therefore, it is harder to neutralize polyclonal antibodies than it is in molecular-targeted therapies, such as anti-epidermal growth factor receptor therapy. BP may be a disease for which anti-idiotypic therapies have potential. The pathogenesis of BP-IgG has been clearly proved using animal models (38, 39). Autoantibodies from more than 80% BP patients target to N-terminal 72 amino acids of the COL17-NC16A domain (40, 41). Furthermore, a precise epitope mapping study showed that 14 amino acids within the NC16A domain are recognized by 50–60% of BP sera (42). These indicate that BP-IgG may be less variable than other autoimmune disorders and may be neutralized effectively by anti-idiotypic antibodies. In conclusion, we demonstrated the effects of IVIg in preventing COL17 depletion induced by BP-IgG due to anti-idiotypic antibodies. This study is the first to demonstrate the presence of anti-idiotypic antibodies against anti-COL17 IgG in IVIg. Disease-specific therapies using anti-idiotypic antibodies may have potential as treatments for BP.

## Ethics Statement

In accordance with the Hokkaido University Hospital bylaws and standard operating procedures approved by the Hokkaido University Hospital Review Board, we obtained patient consent for experimental procedures to be performed at Hokkaido University Hospital. The studies were conducted in accordance with the Helsinki guidelines.

## Author Contributions

YM, MK, ET, and HI performed the experiments. HI, HU, YK, and HS designed the experiments. HI wrote the manuscript, and all the coauthors had final approval of the submission.

## Conflict of Interest Statement

The authors declare that the research was conducted in the absence of any commercial or financial relationships that could be construed as a potential conflict of interest.

## Abbreviations

IVIg, intravenous immunoglobulin; AIBD, autoimmune blistering disease; EBA, epidermolysis bullosa acquisita; BP, bullous pemphigoid; COL17, type XVII collagen; NC16A, the non-collagenous 16A domain; RT, room temperature; BSA, bovine serum albumin; FcRn, neonatal Fc receptor; ELISA, enzyme-linked immunosorbent assay.
